# Rating Scales for Obstructive Sleep Apnea Syndrome: The Importance of a Comprehensive Assessment

**DOI:** 10.7759/cureus.100725

**Published:** 2026-01-04

**Authors:** Bruno Bordoni, Bruno Morabito

**Affiliations:** 1 Physical Medicine and Rehabilitation, Foundation Don Carlo Gnocchi, Milan, ITA; 2 Faculty of Medicine, Università Cattolica del Sacro Cuore Agostino Gemelli University Hospital, Rome, ITA

**Keywords:** continuous positive airway pressure, cpap, diaphragm, obstructive sleep apnea syndrome, osas, rating scale, sleep-disordered breathing, tongue

## Abstract

Obstructive sleep apnea syndrome (OSAS) is characterized by cyclical apnea states during sleep, caused by alterations in the dilatory muscles of the pharynx and tongue. It is an underestimated and underappreciated disorder, which can affect a person's health and quality of life, as well as trigger the onset of several other systemic diseases. Patients diagnosed with OSAS present with clinical pictures that are not always easy to distinguish and may be associated with multiple comorbidities. This heterogeneity makes early diagnosis difficult and complicates not only the resolution of sleep apnea but also the balancing of clinical priorities. Various OSAS assessment and classification scales exist in the literature, as early diagnosis is essential for more effective treatment. This narrative review article reviews the assessment scales for OSAS and briefly discusses the pathologies that may be encountered in the presence of this syndrome. Early detection of OSAS not only means early clinical intervention but also means preventing pre-existing pathologies from worsening.

## Introduction and background

Sleep-disordered breathing (SDB) includes several disorders, such as obstructive sleep apnea syndrome (OSAS), primary snoring, Cheyne-Stokes respiration, central sleep apnea (CSA), sleep-related hypoventilation, and hypoxemia [1]. The most common is OSAS, where functional and structural alterations of the pharyngeal airways cause intermittent apneas, hypopneas, and hypoxia, limiting airflow and preventing proper nighttime rest [2]. It is estimated that approximately one billion people may suffer from OSAS, approximately a third of whom are adults [3].

Historically, OSAS was only classified in 1965. According to the International Consensus Statement on Obstructive Sleep Apnea: “It is defined on the basis of nighttime and daytime symptoms as well as objective data from a sleep study (home sleep apnea test or polysomnography)” [4]. Furthermore, approximately 50% of patients suffer from excessive daytime sleepiness (EDS) [5].

Constant and chronic sleep disruption causes various consequences for the person who suffers from it. OSAS induces alterations in saturation (hypercarbia, hypoxemia), increases systemic oxidative stress, and stimulates the presence of sympathetic hyperactivity [5]. In the long term, OSAS contributes to the onset of multiple disorders and pathologies. Patients may suffer from daytime drowsiness and general fatigue, with less attention in daily and work activities. A negative alteration of memory, cognitive decline, and fluctuations in mood and emotional status are recorded, which can lead to psychological and psychiatric problems [5,6]. There is a bidirectionality between OSAS and the presence of cardiovascular (congestive heart failure), neurological (stroke, dementia), metabolic (obesity, diabetes), oncological and nephrological diseases in this patient population [5,6]. The health-related quality of life (HRQoL) undergoes a decline with increase in the mortality rate [5,6].

The American Academy of Sleep Medicine (AASM) suggests some definitions of the respiratory disturbance events. Apnea is considered as an event that occurs for ≥10 seconds and with a decrease in airflow of ≥90% compared to the baseline value, while hypopnea occurs when there is a desaturation of ≥3-4% compared to the baseline value and for ≥10 seconds [4]. The gold standard for measuring apnea and hypopnea is polysomnography [4]. These measurements allow us to calculate the apnea-hypopnea index (AHI), which allows us to better define the severity of OSAS. AHI is the result obtained by dividing the number of respiratory disturbance events during sleep (apnea/hypopnea) by the number of hours of sleep [4]. AHI can represent both peripheral obstructions (laryngeal area) and obstructions resulting from events in the respiratory centers. An AHI value of 5-15 indicates a mild disorder, a value of 15-30 indicates the presence of moderate OSAS, while over 30 is considered a severe condition [7].

Different approaches in the literature exist to combat and/or improve OSAS. The gold standard and non-pharmacological and non-surgical first-line therapy, is continuous positive airway pressure (CPAP), applied during sleep [8]. Oral aids can be applied to the patient to change the shape and function of the tongue or jaw, such as the tongue retaining device (TRD), to help with lingual protrusion; whereas the mandibular repositioning device (MRD) aims to bring the jaw into greater protrusion [4]. Devices that allow for improved nasal breathing, such as internal and external nasal dilators, can be used [4]. Infiltrating surfactants into the pharyngeal space is a strategy that the clinician could employ to reduce airway friction, as well as prescribing oxygen at night [4,9]. Other approaches include speech therapy and physiotherapy to improve the function of the dilatory muscles of the upper airways [4]. Second-line treatment for patients who are not compliant in using CPAP (less than four hours per night), and/or with problems in the mandibular area (too retruded) is surgery, where the surgeon performs maxillomandibular advancements [4].

Patients diagnosed with OSAS present with clinical pictures that are not always easy to distinguish and with overlapping of multiple concomitant pathologies; this heterogeneity makes early diagnosis difficult and makes not only the resolution of sleep apneas more problematic, but also the weighing of clinical priorities. If the male sex has a gender predisposition for the detection of OSAS (greater predisposition >50 years), the female sex (greater predisposition >65 years) has more recognizable clinical characteristics. For example, women complain more of disorders such as insomnia, anxiety and depression, general fatigue, and morning headaches [5].

Despite the high incidence of OSAS, approximately 80% of patients remain undiagnosed [3,10]. To address this lack of clinical diagnosis, several assessment scales have been identified in the literature, with the aim of early detection of this disorder and/or assessing its clinical burden. If the results are positive, the patient should be referred for further instrumental investigations and/or initiated on a treatment plan to prevent the onset of apnea.

This narrative review highlights the major assessment scales for OSAS in the adult population, and a new scale recently proposed. Being able to identify the presence of this respiratory problem is vital, not only for early application of appropriate therapies, but also for immediate post-surgical care, to avoid prolonged treatment during hospitalization.

## Review

This narrative review searched the literature (PubMed) for the major scientific references on OSAS assessment scales, the most well-known and used, as well as those with the least impact, and evaluated articles with greater statistical weight on the pathologies associated with patients diagnosed with OSAS.

**Figure 1:**
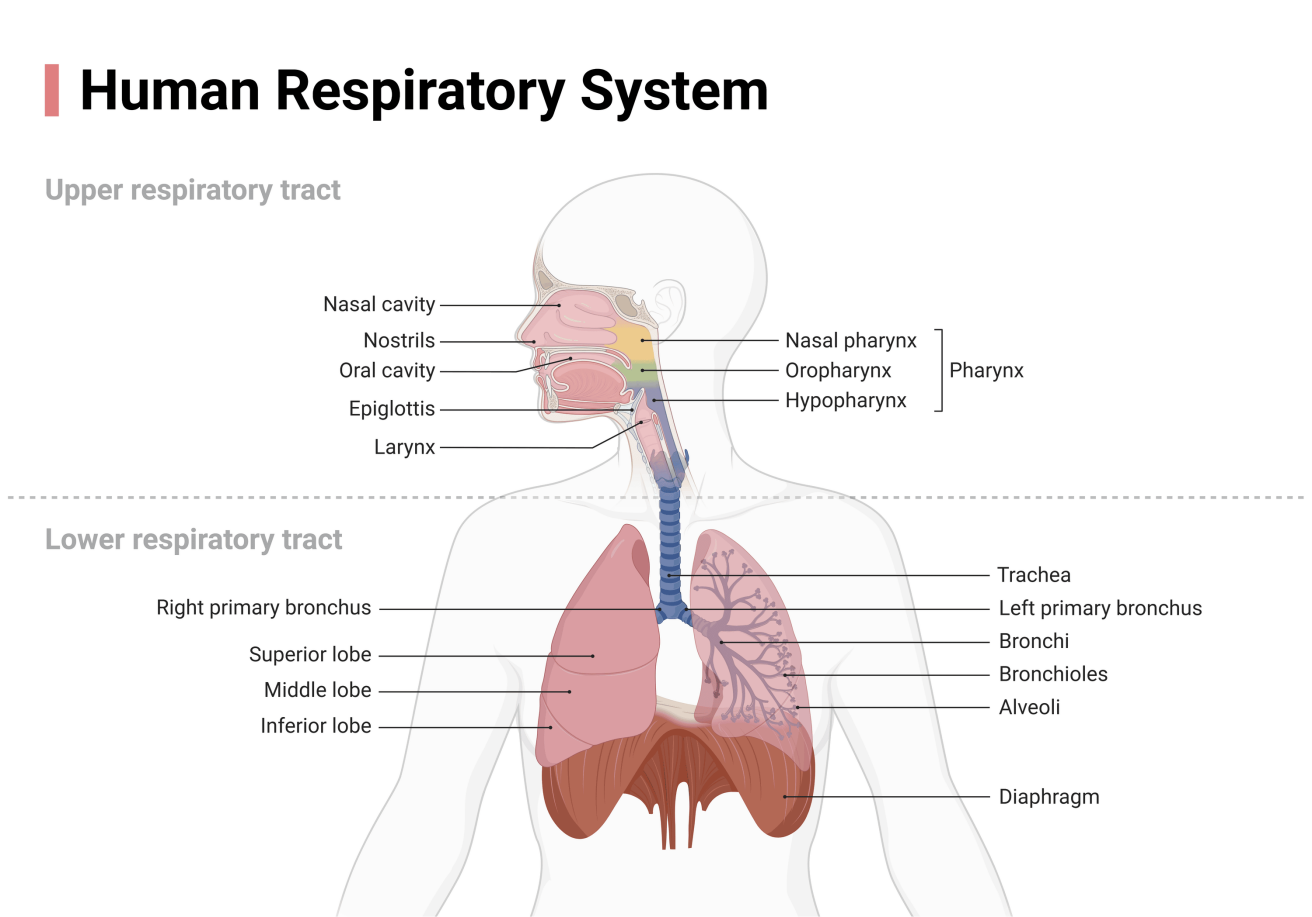
The image schematically illustrates the upper and lower airways Obstructive sleep apnea syndrome (OSAS), due to its chronicity, could cause damage to the respiratory organs. Image credit: Created by Bordoni B. via a subscription on BioRender.com

The curative approach in the presence of OSAS involves multiple clinical figures and medical specializations, from the psychiatrist to the cardiologist, from the maxillofacial surgeon to the dentist, from the neurologist to the pulmonologist, and from the anesthesiologist to the otorhinolaryngologist [4]. Likewise, other figures are involved in non-pharmacological and non-surgical treatment, such as the physiotherapist, speech therapist, and osteopath [11-13]. OSAS negatively influences the upper airways, such as the morphological and functional alteration of the tongue (tongue scalloping) and of the pharyngeal dilator muscles, but also of the lower airways, as alterations in intrathoracic pressure occur, which can damage virtually all the viscera in the thorax (Figure 1) [14-17].

OSAS questionnaires

The Epworth sleepiness scale (ESS) has been validated to detect EDS. EDS not only represents an annoying sensation for the person, but it is also a condition that can be dangerous and induce an increase in mortality due to fatal events while driving or at work [18]. This is even more true if EDS is linked to the presence of OSAS [18]. Despite the importance of daytime sleepiness, there is no valid test to always detect its presence [18]. ESS is a self-administered assessment, and according to the author of the scale, it can identify not only OSAS, but also narcolepsy and idiopathic hypersomnia [19].

The patient is asked to answer whether he/she feels sleepy during the day, compared to the actual sensation of physical tiredness: “How likely are you to doze off or fall asleep in the following situations, in contrast to feeling just tired? This refers to your usual way of life in recent times. Even if you have not done some of these things recently try to work out how they would have affected you. Use the following scale to choose the most appropriate number for each situation” [19]. The score ranges from zero (no sleepiness), 1 (slight sleepiness), 2 (moderate possibility of falling asleep due to sleepiness), and 3 (high probability of falling asleep due to sleepiness) [19]. The patient is asked to give a score in relation to some daily activities (eight in total) and basic already soporific, such as driving a car in traffic or sitting in front of the television [19]. In the original article, it was found that OSAS patients showed a higher ESS score; subsequent works highlighted that patients undergoing CPAP had a lower ESS score [18]. The validity of test administration must be reproducible and reliable. Subsequent studies to compare reproducibility and reliability have highlighted an intra-individual discrepancy in the clinical population, since phenotypic/endotypic variables should be included, which are absent from the ESS [18]. Therefore, the test-retest variability with the administration of ESS is unknown [18]. In clinical practice, the custom has emerged to consider an ESS ≥11 to indicate EDS, but categorical variables are not specified [18,20,21]. For example, based on the sex of belonging, the ESS score varies greatly (pre- and post-CPAP), confounding the results [18]. More specific tests to detect EDS, and in particular narcolepsy, idiopathic hypersomnia, and unspecified EDS are the Multiple Sleep Latency Test (MSLT) and the Maintenance of Wakefulness Test (MWT) [18,22,23].

STOP-Bang questionnaire

The acronym STOP indicates Snoring (how loud is the sound of snoring), Tiredness (evaluates sleepiness), Observed apnea (witness), and high blood Pressure, while the acronym Bang includes body mass index, age (over 50 years), neck circumference (≥40 cm), male gender (are you male?) [24]. The questionnaire consists of eight questions (yes/no), of which four are STOP and four Bang. Generally, a score of ≥3 is a threshold with sensitivity (81% to 99%) and specificity (20% to 60%) in identifying patients with obstructive apnea of the upper airways [24]. The questionnaire minimizes false negative results but not false positives [24]. We found a slightly modified STOP-Bang questionnaire in the literature, which changed the meaning of T, where T includes a question to evaluate whether ESS is less than or equal to 10 [25,26].

Berlin questionnaire

It is another test designed in 1996 and given to the patient for self-assessment in primary care settings, to discern the presence of OSAS. The test has good sensitivity and a very good negative predictive value but is lacking in positive predictive value [27]. It is composed of 10 questions placed in three specific categories. In the first category, it tries to give a value to the extent of snoring, in the second it evaluates the extent of sleepiness during the day, while in the third and final category there is only one question to understand what the blood pressure values, and BMI are [27]. As for the ability to classify patients with OSAS, it is very similar to the STOP-Bang questionnaire [27].

Neck, Obesity, Snoring, Age, Sex (NoSAS)

The Lausanne NoSAS score test uses a score ranging from 0 to 17, where a score equal to or greater than eight could highlight the presence of OSAS [28]. The resulting neck circumference greater than 40 centimeters receives a score of four; the patient receives three points if he or she has a BMI over 25-30 kg/m^2^, or receives five points if the BMI is equal to or greater than 30 kg/m^2^; if snoring is detected the person receives two points; four points if the patient's age is greater than 55 years; if the patient is male, two points are added [28]. NoSAS demonstrates low sensitivity and high specificity when gender is taken into consideration [29]. Furthermore, like other questionnaires, the presence of other comorbidities makes the examined results confounding [30].

Sleep disorders questionnaire (SDQ)

SDQ has a long history of modifications and adaptations, starting in 1983 with the Sleep Questionnaire and Assessment of Wakefulness (SQAW) [31-33]. Compared to the 800 questions of the assessment initially designed, currently only 176 items are used and there is a formulation of five subgroups for the assessment of sleep disorders: narcolepsy; sleep apnea; periodic limb movement disorder; in the presence of psychiatric pathology; sleep disorders related to drug abuse [31]. Probably, it could be dispersive in identifying OSAS. SDQ tries to identify not only OSAS, but also other sleep disorders, determined by International Classification of Sleep Disorders, Third Edition (ICSD-3): insomnia; parasomnia (for example, nightmares that disturb the regular course of sleep); alterations of the sleep/wake cycle; daytime sleepiness; unintentional body movements during sleep; nocturnal respiratory problems [34]. From SDQ, other similar scales are born or simply translated into the language of the country in which it is used clinically [35,36].

Minor questionnaires on sleep disorders

There are several other sleep disorder assessments that are used but are not validated or are too dated, or not specific for upper airway obstruction sleep disorders, such as the Hawaii Sleep Questionnaire (HSQ) [32]. The latter is organized in 16 different areas and over a 100 questions, but is not specific for OSAS. The Pittsburgh Sleep Quality Index (PSQI) is a self-rated questionnaire, which evaluates sleep quality in 10 questions distributed in multiple areas of interest but is not specifically oriented for the detection of the presence of OSAS [37]. The Leeds Sleep Evaluation Questionnaire is formulated as a linear analog scale (100 mm line), built on 10 questions self-administered by the patient, but is not specific for OSAS [38]. The MWT evaluates daytime disturbances resulting from difficulty in sleeping exhaustively, while the Oxford Sleep Resistance Test (OSLER) evaluates altered daytime behaviors resulting from OSAS, associated with another test that measures reaction times in people with sleep problems or the Multiple Unprepared Reaction Time (MURT) [39-41]. Another test like the previous ones is the Sustained Attention to Response Task (SART), created in 1997, which evaluates daytime alertness in people with sleep disturbances, but is not specific for patients with OSAS (Table 1) [42].

**Table 1:** Summary of the rating scales for OSAS OSAS: Obstructive sleep apnea syndrome [18-43].

Name	Objectives
Epworth sleepiness scale (ESS)	ESS has been validated to detect sleepiness during the day (EDS). EES is a self-administered assessment. The score ranges from zero (no sleepiness), 1 (slight sleepiness), 2 (moderate possibility of falling asleep due to sleepiness), and 3 (high probability of falling asleep due to sleepiness).
STOP-Bang questionnaire	The acronym STOP indicates snoring (how loud is the sound of snoring: no=0; yes=1), tiredness (evaluates sleepiness: no=0; yes=1), observed apnea (witness: no=0; yes=1), and high blood pressure (no=0; yes=1), while the acronym Bang includes body mass index (≤35 kg/m²=0; >35 kg/m²=0), age (≤50 years=0; >50 years=1), neck circumference (>40 cm=0; ≤40 cm=1), male gender (female=0; male=1). The higher the score, the greater the likelihood of developing OSAS.
Berlin questionnaire	It is composed of ten questions placed in three specific categories. In the first category it tries to give a value to the extent of snoring, in the second it evaluates the extent of sleepiness during the day, while in the third and final category there is only one question to understand what the blood pressure values, and body mass index.
The Lausanne NoSAS score test (Neck, Obesity, Snoring, Age, Sex)	Lausanne NoSAS uses a score ranging from 0 to 17, where a score equal to or greater than 8 could highlight the presence of OSAS. The resulting neck circumference greater than 40 centimeters receives a score of four; the patient receives three points if he or she has a body mass index over 25-30 kg/m2, or receives five points if the body mass index is equal to or greater than 30 kg/m2; if snoring is detected the person receives two points; four points if the patient's age is greater than fifty-five years; if the patient is male, two points will be added.
Sleep Disorders Questionnaire (SDQ)	Compared to the eight hundred questions of the assessment initially designed, currently only 176 items are used and the formulation of 5 subgroups for the assessment of sleep disorders: narcolepsy; sleep apnea; periodic limb movement disorder; in the presence of psychiatric pathology; sleep disorders related to drug abuse. Probably, it could be dispersive in identifying OSAS. SDQ tries to identify not only OSAS, but also other sleep disorders, determined by International Classification of Sleep Disorders in its third edition: insomnia; parasomnia (for example, nightmares that disturb the regular course of sleep); alterations of the sleep/wake cycle; daytime sleepiness; unintentional body movements during sleep; nocturnal respiratory problems.
Minor questionnaires on sleep disorders: Hawaii Sleep Questionnaire (HSQ)	HSQ is organized in 16 different areas and over a hundred questions but is not specific for OSAS.
Pittsburgh Sleep Quality Index (PSQI)	PSQI is a self-rated questionnaire, which evaluates sleep quality in ten questions distributed in multiple areas of interest but is not specifically oriented for the detection of the presence of OSAS.
Leeds Sleep Evaluation Questionnaire	Leeds Sleep Evaluation Questionnaire is formulated as a linear analog scale (100 mm line), built on ten questions self-administered by the patient, but is not specific for OSAS.
Maintenance of Wakefulness Test (MWT)	MWT evaluates daytime disturbances resulting from difficulty in sleeping exhaustively.
Oxford Sleep Resistance Test (OSLER)	OSLER evaluates altered daytime behaviors resulting from OSAS, associated with another test that measures reaction times in people with sleep problems or the Multiple Unprepared Reaction Time (MURT).
Sustained Attention to Response Task (SART)	SART evaluates daytime alertness in people with sleep disturbances, but is not specific for patients with OSAS.
SANReSP	SANReSP consists of six questions regarding the presence of apnea, snoring, and daytime tiredness, as well as any nocturia and medications taken for hypertension. Patients self-administer the questionnaire.

A recent Italian OSAS assessment scale is the SANReSP, the origin of which is unspecified. The questionnaire consists of six questions regarding the presence of apnea, snoring, and daytime tiredness, as well as any nocturia and medications taken for hypertension. Patients self-administer the questionnaire [43].

Comorbidities present with OSAS

Patients with OSAS (about 80%) present several comorbidities, which increase in percentage with the progression of the severity of the respiratory disorder [42]. Sleep apneas/hypopneas occur mainly during the rapid eye movement (REM) phase of sleep, when the electrical activity of the lingual muscle complex is lower, and where the orthosympathetic autonomic system takes over to a greater extent [44]. This is much more frequent in young patients, with a diagnosis of moderate OSAS, in females; EDS are more common with a more negative impact on the quality of life and a greater incidence of depression [45]. Comorbidities, especially those related to the cardiovascular system, increase the percentage of mortality [46].

Knowing the presence of comorbidities allows us to identify the subgroups of patients with OSAS and better manage the type of treatment. For example, patients with moderate-severe obesity with the use of CPAP seem to suffer less cardiovascular damage; it seems that the effect of CPAP is more effective in males than in females [44].

Presence of cardiovascular disease and/or cardiovascular surgery

Recent data (2025) confirm that OSAS increases the risk of cardiovascular disease and events [47,48]. In patients with acute and/or chronic cardiovascular diseases, such as coronary artery disease, hypertension, heart failure, and atrial fibrillation, OSAS can be diagnosed in approximately 85%, many of whom are not diagnosed before the cardiac event [45].

We are not sure whether OSAS is the main cause or a related event, since the post-CPAP outcome is not the same for all patients.

Chronic hypoxia resulting from this nocturnal respiratory disorder generates a cascade of non-physiological metabolic events such as oxidative stress, which cause an increase in the immune response with a systemic pro-inflammatory environment [44]. Systemic inflammation leads to structural alterations of the arterial and coronary vessels (endothelial dysfunction), chronic intervention of the sympathetic system, and a decrease in the parasympathetic system; an inability of the organism to manage blood pressure (hypertension, particularly nocturnal hypertension) and cardiac remodeling are recorded [44,49].

The increase in upper airway pressures that the patient must overcome during sleep leads to an increase in negative thoracic pressures, with increased venous return. These events induce a preload of the right ventricle, with an increase in blood in the right atrium (increased reservoir function and atrial enlargement) [50]. The right ventricle undergoes remodeling with an increase in end-diastolic volume (volume remaining after a diastole). The result will be a reduced force in systole and increased heart size, with subsequent development of heart failure and pulmonary hypertension [50].

Many patients undergoing cardiac surgery suffer from OSAS, many of whom are diagnosed only after surgery [51]. To help identify patients with pre-surgery OSAS, percentages of which are greatly underestimated, there are some scales, which still have limitations in their results, include the perioperative sleep apnea prediction (P-SAP), DES-obstructive sleep apnea (DES-OSA, which is a morphological test), and the American Society of Anesthesiologists (ASA) list (low sensitivity) [45].

Presence of respiratory pathologies and/or lung surgery

Overlap syndrome (OVS) is the concomitant presence of OSAS and COPD; OVS affects 0.5-1% of the population over 40 years of age [52]. After 65 years of age, OVS increases the risk of mortality [53]. Pulmonary hypertension (PH) is another problem found in patients with OSAS [54]. The percentage of OVS detection varies according to the study taken into consideration, with a variability from 15 to 85% [55]. We do not have much data on lung surgery and OSAS, except for interventions related to lung resection for neoplasms. In the latter context, it seems that the presence of OSAS can increase the occurrence of tumors and recurrences [56,57]. Patients suffering from OSAS may have a 30% increased risk of developing lung tumors, compared to subjects without OSAS [58-60].

Presence of obesity

Obesity is found in patients with OSAS with a high percentage (70%); the greater the accumulated weight, the worse the picture of nocturnal respiratory disorders [45]. In addition to the greater activity of the sympathetic system, there is also a dysfunction of leptin. Leptin (adipokine) is known to have an anorectic effect, and to be increased in obese subjects due to nocturnal hypoxia and a probable resistance to leptin itself [61]. This hyperleptinemia creates a difficulty of respiratory drive with a concomitant reduced hypercapnic response [61]. Obesity also leads to mechanical and structural alterations at the level of the upper airways, such as a reduction of the lumen for the passage of air and a greater collapsibility, leading to chronic apnea/hypopnea phenomena [45]. Furthermore, weight gain prevents the correct movement of the thorax and abdomen, preventing the adequate formation of intrathoracic pressures [45].

Patients undergoing a lung transplant seem to show a 42.9% occurrence of OSAS post-surgery [59]. Patients with non-idiopathic pulmonary fibrosis show a high prevalence of OSAS, with a percentage between 20 and 80% [60].

Presence of diabetes

With an increase in the severity of OSAS, the occurrence of type 2 diabetes increases; equally, the relationship with the presence of hyperlipidemia also increases [45,62]. If there is a close relationship between glycemic values, diabetes, obesity and OSAS, there is a close relationship between OSA, dyslipidemia and the presence of periodic limb movements [63]. The latter event could be linked to an increase in the sympathetic system in this type of patients [63].

Presence of renal pathologies

There is a relationship between OSAS and the presence of renal pathologies, both as a cause and/or as an effect [62]. Probably, the presence of systemic hypertension could damage renal function in a chronic manner, as chronic renal failure [54,62,64,65]. End-stage renal failure involves 57% of people diagnosed with OSAS, and 73% of these show renal pathologies. In patients undergoing dialysis, the presence of chronic hypoxia increases the level of mortality [65].

Cognitive alterations and Alzheimer’s disease

Neurocognitive alterations related to OSAS not only include concentration disorders, but also mood alterations such as depression and dementia. Research reports that patients with OSAS are 1.58 times more likely to develop dementia, compared to subjects without nocturnal breathing disorders [45]. This relationship is independent of age, gender or genetic alterations [45]. There is a relationship between OSAS and the possibility of developing Parkinson’s disease [65]. Pathologies involving the central nervous system are associated with a high percentage of suffering a stroke, or of recording OSAS post-stroke (60-70%) [45,65]. On the other hand, the finding of anxiety and depression in patients diagnosed with OSAS varies from 14.4 to 63% [65,66].

Tumors and OSAS

There is conflicting data in the literature regarding the relationship between the detection of tumors and OSAS. There seems to be a predisposition in patients with OSAS to develop tumors, in particular: renal; prostate; central nervous system; breast; skin (melanoma); colon and rectum; uterus; nose; lungs, pancreas; bladder; larynx [45,58,65]. In the palliative oncology field, there is an evaluation scale, namely, the Oral Symptom Assessment Scale (OSAS), which derives from a previous scale, the Memorial Symptom Assessment Scale [67]. The aim is to intercept obstruction disorders and carve out a better curative approach.

Other disorders in the presence of OSAS

There are many disorders and pathologies associated with the presence of OSAS. Chronic hypoxia causes neuropathies like demyelinating neuropathy, the symptoms of which can be found in 71% of patients [65]. This neuropathy is parallel to the severity of nocturnal saturation [65]. Patients with OSAS and neuropathy can present with central disorders, such as damage to the optic nerve, or peripheral disorders, such as carpal tunnel syndrome [68-71].

Other disorders that are found in patients are glaucoma, floppy eyelid syndrome, retinal vein occlusion, keratoconus, and other ocular pathologies [72-74]. The severity of OSAS also correlates with the presence of idiopathic intracranial hypertension [75].

Approximately 10% of patients suffer from dyspepsia and other gastrointestinal disorders, such as gastroesophageal reflux (GERD), abdominal pain, inflammatory bowel disease, and nonalcoholic fatty liver disease [65,76].

GERD is common with OSAS, as is laryngopharyngeal reflux (LPR) or silent reflux, which is present in approximately 49% of patients with OSAS [77]. GERD and LPR further damage the esophageal, laryngeal, and pharyngeal tissues, which cause reflex alterations allowing the evolution of OSAS (increased collapsibility) [78]. Symptoms related to GERD and LPR in patients are: dry mouth (in 74%), chronic cough, dysphonia; throat clearance, sensation of incomplete swallowing and food residue in the throat, more caudal position of the hyoid bone, and dysphagia [79]. Dysphagia (presbyphagia in older people) is underestimated in OSAS patients, with a finding of up to 78%, as it presents as a subclinical dysfunction [80-82]. GERD, asthma, chronic rhinosinusitis, and OSAS are common and overlapping dysfunctions that are grouped in the chronic cough/asthma, obstructive sleep apnea, rhinosinusitis, and esophageal reflux disease (CORE) syndrome [83]. OSAS patients often present otolaryngological disorders, such as sub-clinical disorders (vertigo, nausea, vomiting, nystagmus) in the vestibular area, especially in patients with moderate-severe syndrome [78,84]. Patients may also suffer from Eustachian tube dysfunction due to chronic altered pressures during snoring, which can lead to hearing loss, retraction of the tympanic membrane, and chronic middle ear otitis [80]. In OSAS, we can find symptoms such as facial pain and headache, along with rhinorrhea, anosmia, otalgia, reduction in the ability to discern odors (lesion of the olfactory nerve), and taste [80,85].

The lingual complex in patients with OSAS is dysfunctional, and an electromyographic examination of the genioglossus muscle is not enough to identify a tongue dysfunction in patients with OSAS [86]. The tongue is thickened and/or has a fallback position [87]. All these altered characteristics favor the occlusion of the upper airways [88]. Although the tongue (if dysfunctional) plays an extraordinary role in sleep apnea syndromes, it is not usual to check the lingual function in a patient, for example with the simple tongue performance test [89].

Vitamin D levels appear to be low in this type of patient, although the reasons are unclear [90,91]. In patients with OSAS, painful somatic areas appear, such as the lumbar area (with a high percentage of lumbar spondylosis), and the cervical area, in approximately 13.8% of all patients [92,93]. Many patients complain of temporomandibular joint disorders, such as pain, decreased mouth opening, joint noises and bruxism; the prevalence of this problem involves 52% of patients with OSAS [94,95]. The phenomenon of nocturia is prevalent in patients, with a finding in 61.7%, and has a greater presence based on the severity of OSAS [45].

Increased estrogen levels may be a concomitant factor for nasal congestion, leading to narrowing of the upper airways, as in pregnancy, particularly from the end of the second trimester [45].

Another problem that is often underestimated is the dysfunction of the diaphragm muscle. A unilateral diaphragmatic lesion is often sub-clinical, and it is not easy to identify its presence. In this context, the OSAS symptoms become more severe [96]. If the diaphragm muscle does not contract correctly, it can become a cause for OSAS [97,98]. Considering that the diaphragm is the main inspiratory muscle, it is governed by the respiratory centers of the upper airways, it would be useful to evaluate how this muscle behaves to identify patients with OSAS, even with a simple Bordoni diaphragmatic test (BDT) [99,100].

Another problem that is frequently encountered in patients with OSAS is polycystic ovary syndrome (PCOS), with a percentage of 17-75% [101]. Probably, there is an overlap with other pathologies, such as diabetes, obesity and hypertension, and it is not easy to discriminate the initial cause of this finding [101].

In conclusion, some risk factors that can exacerbate the symptoms of OSAS or be a source of this disorder are a low HRQoL, due to the disorder itself or for socioeconomic reasons, and the lack of physical activity (Tables 2, 3) [8,101,102].

**Table 2:** Symptoms that can be found in the patient affected by obstructive sleep apnea syndrome (OSAS) [44-98,101]

Symptoms found in patients with OSAS
Depression, anxiety and stress
Lower health-related quality of life (HRQoL)
Increased Body Mass Index (BMI)
Gender (male over 55 years old); female (over 65 years)
Familiarity
Facial morphology (e.g. small jaw)
Kidney disease
Lower vitamin D levels in patients without comorbidities
Cardiovascular (arterial hypertension, coronary artery disease, arrhythmias, ischemic stroke) and respiratory (chronic obstructive pulmonary disease, asthma, pulmonary hypertension) diseases
Gastrointestinal disorders (peptic ulcer, gastroesophageal reflux, chronic liver disease)
Tumors (lung, prostate, kidney, central nervous system, breast, skin (melanoma), colon and rectum, uterus, nose, pancreas, bladder, larynx)
Involuntary movement of the lower limbs
Parkinson's, Alzheimer's disease, dementia
Central and peripheral neuropathy
Idiopathic intracranial hypertension
Otorhinolaryngological disorders (dry mouth, chronic cough, dysphonia throat clearance, dysphagia, dizziness, nausea, recurrent vomiting, nystagmus, facial pain, chronic rhinosinusitis, headache, rhinorrhea, anosmia, otalgia, olfactory nerve damage, taste alteration)
Neuromotor incoordination of the tongue
Somatic symptoms (low back pain, neck pain, temporomandibular joint disorders, bruxism)
Nocturia
Diaphragmatic dysfunction
Polycystic ovary syndrome

**Table 3:** Risk factors that increase the probability of onset of obstructive sleep apnea syndrome (OSAS) [4-6,8,45,101].

Risk factors for OSAS
Smoking
Excess alcohol intake
Excess caffeine intake
Pregnancy
Obesity
Pre-existing chronic pathologies
Cranimetric morphological alterations
Genetic dysfunctions
Sedentary lifestyle
Low quality of life

Preventive questionnaire for OSAS: Pre-OSAS

Pre-OSAS is a new assessment awaiting validation, aimed at early detection of OSAS [103]. It is a clinician-led assessment (not self-administered) that considers the major risk factors and comorbidities present in the anamnesis, with two active motor tests (performance tongue test (PTT) and BDT) [89,99,103]. The latter assess whether the tongue and diaphragm are correctly integrated into an adequate neurophysiological context.

Pre-OSAS consists of 29 items, with a maximum score of 25.5, where seven items have a score of 0.5, and the other 22 items have a score of 1. If the patient's assessment with this scale reaches a score of more than half of the total score (>13), there may be a high percentage of possibility that the patient may suffer from OSAS (Table 4) [103].

**Table 4:** Pre-Obstructive sleep apnea syndrome (OSAS) test The table has an addition (polycystic ovary syndrome*). When multiple symptoms or just one are present in the assessment, the clinician circles the number or checks the box, and the value will always be 0.5. If there is an absence of symptoms or pathologies, or dysfunctions, the clinician leaves the box blank [103].

Preventive questionnaire for OSAS: Pre-OSAS	Score
Snoring	1
Male sex >50 years	1
Female sex >65 years	1
Obesity body mass index >35Kg/m^2^ / Neck circumference ≥40cm	1
Diaphragmatic pathologies/dysfunctions (assessment with the Bordoni diaphragmathic test)	1
Pathologies/Dysfunctions of the lingual complex (assessment with the Tongue performance test)	1
Daytime sleepiness	0.5
Cardiovascular diseases	1
Respiratory disorders	1
Previous thoracic surgeries	1
Diabetes/Metabolic Syndrome	1
Renal disorders	0.5
Liver pathologies	0.5
Depression/Anxiety/Stress	1
Cognitive decline	1
Neurodegenerative diseases	1
Tumors	1
Peripheral neuropathy	0.5
Otorhinolaryngological pathologies/disorders	1
Dysphagia	1
Ocular pathologies/disorders	0.5
Gastrointestinal pathologies/disorders	1
Dysphonia/Chronic dry cough	1
Morphological craniofacial alterations	1
Alterations in taste/smell/hearing	1
Chronic undefined symptoms (dizziness, vitamin D deficiency, nocturia, slowed memory, involuntary movement of the lower limbs at night, dry mouth, facial pain, low back pain, neck pain, bruxism, functional alterations of the temporomandibular joint, headache, gout, polycystic ovary syndrome)*	0.5
Sedentary lifestyle	1
Pregnancy from the third trimester	1
Familiarity	0.5
Total score	

The presence of OSAS can not only cause the onset of new pathologies but also accentuate the symptoms of pre-existing pathologies. For example, patients diagnosed with irritable bowel syndrome (IBS) and sleep apnea may experience an increase in gastrointestinal symptoms when an AHI>30 is found on polysomnography [104]. This means that to help patients with IBS and OSAS, the therapeutic choice should not only focus on the symptoms arising from the intestine, but also on respiratory functions [104]. Furthermore, adults with epilepsy may experience a worsening depression resulting from the presence of OSAS; treating a patient with epilepsy and the aspect of cognitive status means checking the condition of the respiratory airway function when the person is asleep [105].

Early detection of OSAS not only means early clinical intervention but also prevents pre-existing conditions from worsening. Considering the most comprehensive assessment possible, such as pre-OSAS, could help establish a more rapid clinical assessment. Clinicians should likely determine whether the patient suffers from sleep apnea, regardless of the medical specialty or the underlying condition. Further research should be done to adequately distinguish this syndrome, considering the multiplicity of differences between patients [106].

## Conclusions

OSAS is caused by functional and structural alterations of the pharyngeal airways, resulting in intermittent apneas, hypopneas, and hypoxia, limiting airflow and preventing restorative salubrious sleep. Patients diagnosed with OSAS present with clinical pictures that are not always easy to distinguish and may exhibit overlapping comorbidities. This heterogeneity makes early diagnosis difficult and complicates not only the resolution of sleep apnea but also the balancing of clinical priorities. This narrative review article examined the major OSAS assessment and classification scales, emphasizing that early detection of the syndrome not only helps patients accelerate the recovery process (when possible) but also prevents the worsening of apnea-induced comorbidities, prolonging survival.
